# Recent Advances in Protective Vaccines against Hepatitis Viruses: A Narrative Review

**DOI:** 10.3390/v15010214

**Published:** 2023-01-12

**Authors:** Ashraf Elbahrawy, Hassan Atalla, Mohamed Alboraie, Ahmed Alwassief, Ali Madian, Mohammed El Fayoumie, Ashraf A. Tabll, Hussein H. Aly

**Affiliations:** 1 Gastroenterology and Hepatology Unit, Department of Internal Medicine, Al-Azhar University, Cairo 11884, Egypt; 2 Gastroenterology and Hepatology Unit, Department of Internal Medicine, Mansoura University, Mansoura 35516, Egypt; 3 Gastroenterology Unit, Department of Internal Medicine, Sultan Qaboos University Hospital, P.O. Box 50, Muscat 123, Oman; 4 Department of Internal Medicine, Faculty of Medicine, Al-Azhar University, Assiut 71524, Egypt; 5 Microbial Biotechnology Department, Biotechnology Research Institute, National Research Center, Giza 12622, Egypt; 6 Egypt Center for Research and Regenerative Medicine (ECRRM), Cairo 11517, Egypt; 7 Department of Virology II, National Institute of Infectious Diseases, Toyama1-23-1, Shinjuku-ku, Tokyo 162-8640, Japan

**Keywords:** vaccine, HAV, HBV, HCV, HDV, HEV, viral hepatitis

## Abstract

Vaccination has been confirmed to be the safest and, sometimes, the only tool of defense against threats from infectious diseases. The successful history of vaccination is evident in the control of serious viral infections, such as smallpox and polio. Viruses that infect human livers are known as hepatitis viruses and are classified into five major types from A to E, alphabetically. Although infection with hepatitis A virus (HAV) is known to be self-resolving after rest and symptomatic treatment, there were 7134 deaths from HAV worldwide in 2016. In 2019, hepatitis B virus (HBV) and hepatitis C virus (HCV) resulted in an estimated 820,000 and 290,000 deaths, respectively. Hepatitis delta virus (HDV) is a satellite virus that depends on HBV for producing its infectious particles in order to spread. The combination of HDV and HBV infection is considered the most severe form of chronic viral hepatitis. Hepatitis E virus (HEV) is another orally transmitted virus, common in low- and middle-income countries. In 2015, it caused 44,000 deaths worldwide. Safe and effective vaccines are already available to prevent hepatitis A and B. Here, we review the recent advances in protective vaccines against the five major hepatitis viruses.

## 1. Introduction

Viral hepatitis remains an important challenge to human health, as it is a primary cause of death worldwide [1]. Among the causes of acute viral hepatitis, acute hepatitis A virus (HAV) infection has caused the heaviest burden. In the majority of low-income regions, including sub-Saharan Africa and parts of south Asia, the prevalence of HAV antibodies (anti-HAV) may exceed 90% by the age of 10 years [2]. In contrast, the prevalence of anti-HAV is very low (<50% by the age of 30 years) in high-income regions [2]. The anti-HAV seroprevalence shows a mix of intermediate and low prevalence in the middle-income regions of Asia, Latin America, Eastern Europe, and the Middle East [2]. Before the introduction of vaccination, HAV infection had become a major cause of fulminant hepatic failure (FHF) and a significant indication of liver transplantation among children in Argentina [3], Brazil [4], the Republic of Korea [5], and India [6].

Hepatitis E virus (HEV) is another leading cause of acute hepatitis, causing an estimated 3.3 million cases of acute hepatitis each year [7]. The spread of HEV genotypes 1 and 2 is hyperendemic in several countries located in Central, South, and Southeast Asia [8]. Moreover, HEV genotypes 1 and 2 are hyperendemic in East and West Africa, as well as in Mexico [8]. HEV is also endemic in several regions, such as the Middle East, South America, and Singapore. In developed countries, sporadic HEV genotypes 3 and 4 are increasingly being diagnosed [9]. HEV-1, -2, -3, and -4 genotypes can cause acute hepatitis with self-limited evolution, as well as acute liver failure [10]. Pregnant women in the third trimester have a mortality rate of up to 20% when infected with HEV genotype 1, especially in northern India [11]. Furthermore, infection with genotypes 1 and 2 during pregnancy increases the risk of miscarriage, premature birth, or stillbirth [12]. Genotypes 3 and 4 are more commonly associated with neurological disorders [10].

However, infections with hepatitis B, C, or D viruses (HBV, HCV, or HDV) are important causes of chronic hepatitis, liver cirrhosis, and hepatocellular carcinoma (HCC). Approximately 296 million people were living with chronic HBV infection in 2019 [13], and chronic HCV infection constitutes another global health problem, affecting >58 million people worldwide [14]. The global all-age chronic HBV prevalence was 4.1% in 2019. The highest prevalence was in the Western Pacific region (7.1%), followed by the African region (6.5%). In contrast, the lowest prevalence was 1.1% in the European region [15]. The estimated global pooled prevalence of HDV is 0.80% in the general population and 13.02% among HBsAg-positive carriers. HDV is highly prevalent in central Asia, eastern Europe, tropical and central Latin America, as well as central and west sub-Saharan Africa [16]. The global prevalence of viremic (ribonucleic acid-positive) HCV infection is decreasing and was estimated to be 0.7% at the beginning of 2020. In 2020, most viremic infections were concentrated in lower middle-income countries (26.0 million) and upper middle-income countries (19.8 million). The number of viremic infections remained constant or decreased between 2015 and 2020 in all regions, except eastern sub-Saharan Africa [17].

Protective vaccination for hepatitis viruses remains the cornerstone of public health policy to prevent its related morbidity and mortality. It also represents a vital component of the global viral hepatitis elimination response [18]. Hepatitis A (HA) vaccine is highly effective in preventing clinically apparent disease. Despite being vaccine-preventable, HAV outbreaks have occurred among patients infected with the human immunodeficiency virus (HIV) and men who have sex with men (MSM) [19]. In spite of several trials to establish an effective treatment to eliminate HBV, this goal has not yet been achieved [20]. The WHO emphasizes that prevention of the vertical transmission of hepatitis B (HB) is pivotal in reducing the HBV burden. Once achieved, elimination of vertical transmission will be a vital step toward the elimination of HBV [21]. It is worth noting that immunization against HBV will also protect against the coinfection and spread of its satellite, HDV. Although patients with chronic HCV can gain virological cure with direct antiviral agents (DAA), the rate of long-term complications was not related to virological cure among patients with liver cirrhosis. In fact, these patients remain in need of liver transplantation [22]. The protective effect of DAA on HCC recurrence is unclear, where the recurrence rate remains high [23]. Hence, safe and effective protective vaccines are necessary for the complete control and eradication of the HCV epidemic. Although a protective vaccine to prevent HEV infection has been developed and licensed in China, it is not available globally.

The aim of this review is to clarify both the clinical impact of protective vaccines in controlling the five major hepatitis viruses, as well as the frontiers of research in hepatitis vaccines.

## 2. Paper Selection Methodology

The authors performed a literature search focused on protective vaccines against the five major hepatitis viruses. This research was conducted by searching PubMed and Google Scholar databases for articles published from 1975 to December 2022. The publications focusing on candidate hepatitis virus vaccines, vaccination coverage, vaccine efficacy, vaccine safety, long-term vaccine protection, impact of immunosuppressants on vaccine efficacy, causes of vaccine failure, and recent advances in protective vaccines against hepatitis viruses were reviewed and essential data are summarized in this review.

## 3. HA Vaccine

In developing countries, ≥50% of the population has already been exposed to HAV infection by the age of 15 years [1]. Infection with HAV is an increasing concern in the western world, due to travel and immigration from endemic areas. The decline of infection in early childhood because of improved sanitation has resulted in an increasing risk of infection and outbreaks among the adult population [24]. The WHO recommends against large-scale vaccination of populations in highly endemic regions where almost all children contract asymptomatic HAV infection [25]. In contrast, the WHO recommends targeted vaccination programs for high-risk groups in very low-prevalence regions and universal vaccination in intermediate endemic regions [25].

### 3.1. HA Vaccine Candidates

Based on well-conducted, randomized clinical trials on children in the 1990s [25], an inactivated HAV vaccine has been licensed and is available worldwide. This HAV vaccine is available in pediatric (age >1 year) and adult forms and approved for intramuscular injections in two doses with a 6-month interval. Another live attenuated vaccine is licensed in China, India, Guatemala, The Philippines, and Thailand. This vaccine is recommended for all aged 18-months and older. Th live attenuated vaccine is administered as single subcutaneous dose.

### 3.2. Immune Response to HA Vaccine

There are no large-scale studies from different geographic regions comparing the safety and immunogenicity of both inactivated and live attenuated HA vaccines. However, similar safety and efficacy of both vaccines was reported, with a higher seroconversion rate after a single dose of the live attenuated vaccine [26]. In fact, immunization with a single dose of the inactivated and live attenuated HA vaccines resulted in seroconversion rates of >90% and 94%, respectively [25,26]. The protective immunity after the first dose of the inactivated HAV vaccine has been reported to be long term, probably providing life-long immunity. Approximately 80% of vaccines with the live attenuated HA vaccine have documented anti-HAV IgG titers after 15 years. Failure of HA vaccine immunization is extremely rare [27].

### 3.3. HA Vaccine in Travelers

An HAV incidence of 6.0–30.0 cases per 100,000 persons/month is reported in nonimmune travelers to risky destinations. HA vaccination is recommended for travelers aged ≥1 year visiting areas with intermediate or high risk and particularly for immunosuppressed patients, patients with chronic liver disease (CLD), and MSM [28,29]. Remarkably, the inactivated HA vaccine is safe and well tolerated in these patient groups [25,29]. 

The number of HAV cases in developed countries has decreased substantially over the past two decades. This is attributed to the vaccination of travelers against HAV, integration of the HA vaccine into childhood immunization programs, and improvement of sanitation [29].

### 3.4. HA Vaccine in MSM

Periodic HAV outbreaks occur among MSM in developed countries. The most effective approach to control these outbreaks is through HA vaccination [19]. Preemptive and selective reactive HA vaccination is the best strategy to control future outbreaks. Furthermore, testing of HAV seroprevalence among MSM is required to ensure protective immunity [19].

### 3.5. HA Vaccine and Biological Therapy

HAV-seronegative patients with autoimmune diseases are at risk of contracting HAV and are recommended for HA vaccination [30]. There is a lack of data on vaccine efficacy; in fact, a single dose of HA vaccine may not afford sufficient immunization in patients with rheumatoid arthritis [31] and patients using immunosuppressive or biological drugs [32]. Therefore, a second HA vaccination dose after 6 months and testing of postvaccination antibody titers is recommended.

### 3.6. HA Vaccine in Patients with CLD

Patients with CLD are not at an increased risk of contracting HAV infection. However, they are at high risk of developing FHF and acute-on chronic liver failure [33]. HA vaccination is recommended for all patients with CLD in high-income countries. This includes patients with HBV, HCV, liver cirrhosis, nonalcoholic fatty liver disease, alcoholic liver disease, or autoimmune hepatitis [34,35]. The inactivated HA vaccine is well tolerated in patients with mild-to-moderate CLD [36] and should be administered as early as possible [37,38].

### 3.7. HA Vaccine in People with HIV

Up to 87% of persons with HIV infection are at risk of contracting HAV infection due to poor response to HA vaccine or lack of previous immunization. In addition, they are at higher risk of contracting severe HAV infection than people with HAV infection alone. In fact, HIV-infected patients have poor responses to HA vaccine and carry a higher long-term seroreversion rate [39,40]. The risk factors for the poor response to HA vaccine in HIV-infected patients include obesity, high viral load, older age, and HCV coinfection [39,41,42,43,44,45,46,47,48,49,50,51,52,53,54,55]. Two doses of HA vaccine are recommended for people living with HIV to achieve sustained seroresponse rather than single dose [56].

## 4. HB Vaccine

HBV infection is a worldwide leading cause of morbidity and mortality [57]. The WHO global estimates in 2019 revealed that there were 296 million HBV carriers, 1.5 million new infections per year, and an annual mortality of 820,000 individuals [57,58,59,60]. A major pillar of the WHO strategy for the eradication of HBV is the universal HB vaccination [59,60].

### 4.1. HB Vaccine Candidates

The first commercially available HB vaccine was developed in 1982 [61]. The manufacturing technology depended on the extraction of HB surface antigen (HBsAg) from healthy carriers [62]. Despite its efficacy, there was always a theoretical risk of blood borne infection. This limitation stimulated the search for an alternative that resulted in the development of the recombinant HB vaccine [62]. All the genotypes of HBV share an “*a*” antigenic determinant. DNA recombination technology was implemented in yeast to synthesize and assemble an “*a*” antigenic determinant-like protein that was approved by the Food and Drug Administration (FDA) in 1986 [63]. The recombinant HB vaccine provides subtype cross-protective immunity. A series of three doses of recombinant vaccine can elicit long-term protection for more than 30 years [61].

In November 2017, the United States of America (USA) FDA approved the two-dose HB vaccine Heplisav-B^®^. Heplisav-B^®^ uses recombinant HBsAg along with cytidine phospho-guanosine oligonucleotide (CpG 1018) derived from bacterial DNA as an adjuvant [64]. This activates the immune system to initiate a direct response to HBsAg through the toll-like receptor-9 pathway, rather than the multi-immunostimulatory pathway induced by the conventional three-dose vaccine [64].

### 4.2. Efficacy of HB Vaccine

The protective impact of HB vaccine is measurable in different manners, including the efficacy of the vaccine in mounting protective HB surface antibodies (anti-HBs) in vaccinated individuals and the effectiveness in preventing vertical and horizontal transmission. Multiple trials exploring the immunogenicity of Heplisav-B^®^ against recombinant vaccine have concluded that the two-dose Heplisav-B^®^ induced higher immunity in healthy individuals than the conventional three-dose recombinant vaccine [65,66,67]. In fact, the immune response (anti-HBs ≥ 10 mIU/mL) to three doses of Heplisav-B^®^ vaccine reached almost 100% as opposed to approximately 93% elicited by the recombinant vaccine [68].

The risk of vertical transmission for HB envelope antigen (HBeAg)-positive and HBeAg-negative mothers is 70–90% and 10–40%, respectively [69]. The administration of HB birth dose (HepB-BD) vaccine, followed by at least two additional doses of vaccine within 6–12 months [70], is 90–95% effective in preventing vertical transmission [71,72].

In Taiwan, 30 years after the implementation of the national HB vaccination program, the HBsAg-positive rate decreased from 9.7% in university students born before June 1974 to <1.0% in students born after 1992 [73]. Since the introduction of the HB vaccine in the 1990s, there has been a significant decline in the incidence of HBV infection in the USA, especially among children aged <15 years [74]. In particular, the global adoption of HB vaccination decreased the global prevalence of HBV in children aged <5 years from 4.7% to 1.3% in 2015 [62]. The safety of all types of HB vaccines is similar and has been reported in a myriad of studies [68,75,76].

### 4.3. HB Vaccine Failure

The pathogenic mechanism behind the failure of immune response to HB vaccine is the reduced T-cell activation or the failure of T cells to recognize HBsAg [77]. Globally, 10% of HB-vaccinated individuals fail to mount effective immunity. Older age, obesity, smoking, diabetes mellitus, chronic kidney disease, and immune suppression have all been linked with poor response to HB vaccination [77,78,79,80,81,82,83,84,85]. Furthermore, HBV variants with amino acid polymorphisms in the antigenic region (*a* determinant) of small HB surface (S-HBs) protein may infect even vaccinated individuals [86,87,88,89,90,91,92]. The amino acid polymorphisms in these variants are considered to be responsible for the evasion of neutralization of the virus by the anti-HBs induced by the current S-HB vaccine and are designated as vaccine-escape mutations (VEMs).

The immune response to HB vaccination can be further augmented in nonresponders by different interventions, including using additional booster doses, increasing the vaccine dose, use of intradermal route, use of tri-antigen HBV (3A-HBV) vaccine, or use of the large HB surface antigen (L-HBsAg) vaccine [80,93,94,95,96].

The 3A-HBV vaccine contains three HBV surface antigens, viz., pre-S1, pre-S2, and S, unlike currently available HB vaccines that contain only S-HBs. The pivotal phase III study PROTECT demonstrated that this 3A-HBV vaccine is highly immunogenic for adults, including older adults and those with well-controlled chronic diseases [95]. The 3A-HBV vaccine may provide more opportunities for the immune system to respond with anti-HBs, helping the host overcome the limitations of the current S-HB vaccine.

An HB vaccine using the L-HBsAg was recently established [96]. This vaccine induces the anti-HBs capable of neutralizing HBV with VEMs.

### 4.4. Durability of the Immune Response to HB Vaccine

The primary anti-HB protection after HB vaccination lasts for more than two decades in the vast majority of healthy individuals [97]. Nevertheless, the durability of the immune response in immunocompetent individuals is affected by several variables, including age, body mass index, sex, smoking, and diabetes mellitus [98,99,100].

### 4.5. HB Vaccine and Biological Therapy

Biological therapy involves treatment with natural molecules made by the body or prepared in a laboratory. It includes treatment with monoclonal antibodies, adoptive cell transfer, gene therapy, treatment with cytokines, cancer vaccines, oncolytic viruses, immunoconjugates, and the use of targeted therapy [101]. Protection against HBV infections through vaccination is extremely important in patient groups using biological drugs. The immune response to HB vaccination in patients under biological therapy is not only suboptimal, but the average anti-HB titer level in responded patients is also lower than that in healthy controls [102]. Moreover, there have been only marginal improvements in the immune response rate after the use of double-dose vaccination [102].

The immune response rate to HB vaccination significantly decreases in patients receiving tumor necrosis factor-alpha (TNF-α) blockers [103,104]. Ravikumar et al. reported that the response rate to HB vaccination had decreased to 33% in patients receiving etanercept therapy [104]. Moreover, only 1 of 25 patients with rheumatoid arthritis and 3 of 27 patients with spondyloarthritis receiving a TNF-α blocker (infliximab) had responded to HB vaccination [103]. In a more recent study, the overall HB vaccination immune response rate was 53.2% in patients receiving TNF-α blockade therapy. Remarkably, the use of TNF-α blockers was a predictor of poor immune response to HB vaccination in patients with inflammatory bowel disease [105].

Altogether, these data suggest that HB vaccine should be administered before the initiation of biological therapy. Ideally, those patients can be provided an accelerated regimen of HB vaccination to achieve early seroconversion.

### 4.6. Role of HB Vaccine in the Prevention of HCC

Worldwide, there has been a decrease in the incidence of HB-related HCC after the introduction of HB vaccination [106]. This was documented in real-world data from both high- and low-endemic areas.

A significant decrease in the incidence of HB-related HCC was also reported in Taiwanese children [107]. The incidence rate of HCC in children aged 6–14 years declined from 0.70 per 100,000 during 1981–1986 to 0.36 per 100,000 during 1990–1994 [107]. More recent data, reported up to 2017, have demonstrated that the cohort aged ≤30 years, who received the universal infant HB vaccination had a 36% reduction in HCC incidence compared with that before the universal HB vaccination [108].

The impact of HB vaccination on the incidence of HB-related HCC has also been demonstrated in low-endemic countries. A study from Alaska on individuals aged <20 years, who received infant HB vaccination, demonstrated a decline in the incidence of childhood HCC to 0 cases per 100,000 population from a peak incidence rate of 3 per 100,000 before vaccination [109].

### 4.7. HB Vaccination and the COVID-19 Pandemic

The expanded program of immunization attendance and the average monthly immunizations were reduced during the COVID-19 pandemic. This reduction was particularly noticeable for the HepB-BD vaccine [110]. In fact, the COVID-19 pandemic resulted in increased home births, limiting the administration of the HepB-BD vaccine [111]. HB vaccination campaigns have also been halted due to disruptions in the supply chain. Low- and middle-income countries particularly faced with a shortage of HB vaccines during the pandemic [112,113]. In fact, sub-Saharan Africa had breakdowns in the cold chain and limited financial support [114].

The reduced availability and implementation of HB vaccination during the COVID-19 pandemic will have a deleterious impact on the incidence of HBV infection in the new generations. Moreover, it will impede the response of countries to the WHO hepatitis elimination goals by 2030 [114].

### 4.8. HB Vaccination Coverage

The rate of HB vaccination had increased globally from 30% in 2000 to 85% in 2019. However, African nations have recorded a low vaccination coverage of 73%. Moreover, only 43% of African newborns received the HepB-BD vaccine [115]. Similarly, lower global vaccination coverage rates were reported among adults aged >19 years [116], intravenous drug abusers [117], patients with CLD, and those with diabetes mellitus [116].

The WHO emphasizes that universal HB vaccination is pivotal in reducing HBV incidence and eliminating viral hepatitis by 2030 [118]. The WHO Global Health Sector Strategy on Viral Hepatitis has set the target of achieving 90% HB vaccination coverage, including birth dose, by 2030 [119]. A global model of HBV elimination [120] estimated that without a change in the vaccination programs worldwide, there would be 25 million new cases of chronic HBV infection between 2015 and 2030. Based on this modeling study, the WHO metrics to decrease HBV-related morbidity and mortality can only be achieved on a global scale by implementing universal infant HB vaccination [120].

Increasing global infant vaccination coverage to 90% would avoid 4.3 million incident chronic HBV infections and prevent 1.1 million HB-related deaths by 2030. In addition, it is estimated that improving the coverage of infantile vaccination to 90% in combination with a HepB-BD vaccination coverage of 80% would prevent 1.5 million deaths due to HCC by 2030 [120].

### 4.9. HepB-BD Vaccine

In 2009, the WHO recommended that all newborns receive the timely HepB-BD vaccine, irrespective of the mother’s serological status. By 2019, only an estimated 43% of newborns had received it, and only 110 countries were administering the universal HepB-BD vaccine as a routine policy [121].

It was estimated that scaling up the timely HepB-BD vaccination coverage to 90% by 2030 will result in immediate reductions in incident chronic HBV cases. In fact, it would avoid 41,000,000 chronic infections between 2020 and 2100 and will result in 710,000 fewer deaths among those born between 2020 and 2030 worldwide [122].

Delays in the scale-up of the timely HepB-BD coverage will result in both delays in the elimination of HBV and an increase in HBV-related deaths in the coming decades. The challenges to the scale-up of the timely HepB-BD coverage were attributed, in part, to the circumstances created by the COVID-19 pandemic [121]. Home births, especially in rural settings, present a challenge to vaccinating newborns in this time-critical manner. Another challenge is in vaccine transportation to endemic areas regarding the need for cold chain storage; the birth-dose vaccine should be stored between 2 °C and 8 °C.

### 4.10. Cost-Effectiveness of HB Vaccination

A recent USA-based study reported that HB vaccination was more cost effective than the test and treat strategy. The study compared the incremental cost-effectiveness ratios (ICERs) of vaccination versus the screening and treating strategy and found that vaccination was more cost effective. In fact, vaccination had ICERs of $6000 per quality-adjusted life-year gained versus $21,000 for the screen and treat strategy [123].

Universal vaccination was found to be cost effective in areas of intermediate/high HBV endemicity, whereas it was ineffective in areas with low endemicity. Cost-effectiveness had been reported in Iran [124], China [125], and Vietnam [126], as opposed to the UK [127]. Despite being a high-value investment, adopting a universal vaccination strategy consistent with WHO goals could be simpler, less expensive, and less likely to miss children at risk for HBV infection than the selective vaccination strategy [128].

## 5. Hepatitis C Vaccine

HCV was first suspected in 1975 by Alter et al. when they suspected a non-A non-B viral hepatitis [129]. Subsequently, it was confirmed to induce parenteral transmission [130], and its genome was identified in 1989. HCV antibody (anti-HCV) was also discovered by the same group and a diagnostic assay was developed [131]. Since then, several scientists have been discussing the obstacles to develop an effective vaccine for HCV, especially the lack of protection by the developed anti-HCV after the first infection [132].

### 5.1. Hepatitis C Vaccine Candidates

Trials to develop an HC vaccine started in the early nineties using viral vectors to induce viral proteins (glycoprotein) that can stimulate the immune system to develop T-cell response against HCV [133]. Although the initial results were encouraging [134], several vaccines tested over the past decades failed to reach approval for clinical use. There are two major categories of different HC vaccines in development, preventive vaccines and therapeutic vaccines [135].

Preventive vaccines include HCV-like particle vaccines, recombinant protein vaccines, DNA vaccines, peptide vaccines, and viral vector vaccines, or a combination of these [136,137]. Some therapeutic vaccines against HCV have been investigated or are still in development (NCT02772003, NCT01055821, NCT02027116, NCT00606086, NCT00124215, NCT01701336, NCT01094873, and NCT04318379); however, none of them have reached Phase III (Table 1) [138]. When available, an HC vaccine can confer protection to high-risk groups, such as people who inject drugs, patients receiving frequent blood product transfusions, healthcare workers, and people with high-risk sexual behavior, in addition to communities with high prevalence and endemic HCV.

### 5.2. Barriers for Having an Effective Hepatitis C Vaccine

Evidence suggests that HCV infection can be spontaneously cleared in 25–40% of infected individuals because of the interaction between the virus and both innate and adaptive immune systems. Patients clearing the virus spontaneously have a better chance (approximately 80%) to clear it again if reinfected. Although the initial infection was not protective against subsequent infections, this anamnestic immune response encouraged the development of vaccines against HCV [139]. HC vaccines aim either to induce a robust T-cell response or to induce neutralizing anti-HCV [140]. There are numerous barriers/obstacles to developing an effective HC vaccine, including high variability in the genetic structure of HCV due to several mutations that appear in each cycle of replication [141]. These mutations result in the presence of several known genotypes (seven) and several unclassified subtypes [142]. Other obstacles for the development of an effective HC vaccine are the paucity of available experimental animal models to investigate the effect of the vaccine and the poor understanding of the protective immune response to the vaccine [143].

### 5.3. Hepatitis C Protective Nucleic Acid Vaccine

Nucleic acid vaccines, including DNA and RNA vaccines, exhibit promising potential in targeting HCV. Nucleic acid vaccines provide a number of potential advantages over conventional vaccines, including the stimulation of both B- and T-cell responses, improved vaccine stability, absence of any infectious agent, and the relative ease of large-scale manufacture [144]. Moreover, the immune responses induced by nucleic acid vaccines targeted a selected antigen in the pathogen.

Plasmids that comprise sequences encoding a number of proteins or epitopes of HCV are capable of inducing cellular and humoral immune responses in animal models when used intramuscularly or subcutaneously. Studies have reported that the use of plasmids containing structural or nonstructural genes of HCV in animal models can stimulate the response of viral T-specific cells [145,146]. It has also been demonstrated that the use of cytokines, such as interleukin-2 (IL-2), granulocyte macrophage–colony-stimulating factor (GM-CSF), interferon-alpha (IFN-α), and Flt-3 ligand (Flt-3 L) as an adjuvant with a vaccine can protect mice against the expression of tumor antigens induced by HCV [147].

Charles M. Rice, in his Nobel lecture, stated that he hoped we can learn from all the efforts that are being put into developing COVID-19 vaccines [148]. Nevertheless, several hurdles remain to apply all the knowledge gained from COVID-19 vaccines to accelerate HC vaccine development. In fact, the biology and immunology of HCV differ significantly from those of the severe acute respiratory syndrome coronavirus 2 (SARS-CoV-2). However, the as-yet unexplored possibility of an HC mRNA-based vaccine could certainly benefit from the experience and developments in the field of RNA-based vaccines against SARS-CoV-2 [144].

### 5.4. Hepatitis C Vaccine Immunogenicity

Considering the small number of tested HC vaccines in human beings and the small numbers of vaccinated volunteers, it is not reasonable to give solid conclusions concerning the safety and immunogenicity of any of them. However, the initial results are assuring in some trials and raising concerns in others. Frey et al. evaluated the safety as well as the immunogenicity of a novel HC vaccine (HCV E1E2/MF59C.1) administered to three groups of volunteers with different doses (20, 40, or 100 µg) via intramuscular injections at weeks 0, 4, 24, and 48. The vaccine was well tolerated with few nonrelated severe adverse events and with accepted mild-to-moderate adverse events. The vaccine induced both antibody responses and lymphoproliferative responses [149]. Another trial tested an HC peptide vaccine (IC41) on 54 volunteers and reported vaccine-related adverse events in 48% of patients. Some of the adverse events led to withdrawal from the study. Although this vaccine induced >60% response in tested individuals with CD4+ T-cell proliferation, it is advisable to wait for large trials on humans to judge the safety and immunogenicity of these new vaccines [150].

### 5.5. Hepatitis C Vaccine Dose

The majority of tested HC vaccines are administered in frequent, separated doses before evaluating the induced immune response [151,152,153]. However, a single-dose vaccine using hepatotropic adenovirus was able to induce a robust T-cell response in the liver of studied animals [154].

## 6. Hepatitis E Vaccine

HEV is one of the major infectious causes of acute liver dysfunction, representing a global health problem [7]. Moreover, HEV infection can cause chronic infection in immunocompromised persons [155]. Currently, there are eight genotypes of HEV [156]. Genotypes 1 and 2 are only known to cause human illness, mostly transmitted by contaminated water, and they frequently cause significant outbreaks in underdeveloped countries. However, in developed countries, most cases are due to genotypes 3 or 4 through zoonotic transmission.

Due to the lack of efficient cell cultures, various technologies were applied to generate recombinant proteins as potential immunogens for vaccine production. In fact, the HEV genome is a positive-stranded RNA consisting of a short 5′ noncoding region capped with three open reading frames (ORFs) that code for nonstructural proteins (ORF1), capsid proteins (ORF2), and a phosphoprotein (ORF3) [157]. The capsid protein encoded by ORF2 (pORF2) appears to be the ideal candidate for an HE vaccine. Thus, a recombinant HEV capsid protein that can self-assemble into a virus-like particle (VLP) and retain the antigenicity of the native HEV virion was generated [158].

The HEV capsid contains three major domains, viz., S, P1, and P2. The outermost P2 domain (also designated as the E2s domain) appears to be the core antigenic region of pORF2 harboring the majority of key neutralization sites, some of which are essential for virus–host interactions and are responsible for the induction of neutralizing antibodies [159].

### 6.1. Hepatitis E Vaccine Candidates

The first report of an HE vaccine candidate was published in 1993 by scientists at the Centers for Diseases Control and Prevention. This was based on the pORF2 from a genotype 1 HEV expressed in *Escherichia coli* [160]. Subsequently, two VLP-based vaccines against HEV, both containing P2 (E2s) domains, were developed and subjected to clinical trials. The first candidate, a 56-kDa baculovirus-expressed vaccine, rHEV, was developed by GlaxoSmithKline (GSK) [161]. The second vaccine is a further truncated version of pORF2, designated as HEV 239 [162], which was expressed in an *E. coli* system. Then, another, more recent, HEV vaccine candidate, the p179, was developed based on the 439–617 region of ORF2 of a genotype 4 strain expressed in *E. coli* [163].

### 6.2. Hepatitis E Vaccine Efficacy in Real-World Data

Both genotype 1-derived recombinant vaccines, rHEV and HEV 239, have demonstrated efficacy and safety in preclinical trials in monkey models. In clinical trials, the rHEV vaccine was proceeded into an open Phase I trial [164], which demonstrated high efficacy with good tolerability and safety of this candidate vaccine. Subsequently, a double-blind, placebo-controlled Phase II clinical trial of rHEV vaccine was conducted in Nepal, in which 2000 participants randomly received three doses of rHEV vaccine or placebo [161]. Although the results were promising, GSK stopped its development the 56-kDa vaccine largely due to commercial reasons [165], particularly with the appearance of a competitive HE vaccine manufactured using a less expensive bacterial expression technology (HEV 239).

In the same context, HEV 239 was the most extensively studied vaccine and was shown to be safe and effective; however, more data are necessary to recommend its use on a global basis. It has been evaluated through Phase I, Phase II, and Phase III clinical trials in China. Data from these clinical trials suggest that the HEV 239 vaccine is well tolerated with no associated serious adverse events. In the Phase I trial [166], 44 healthy seronegative adults received two doses, the vaccine was well tolerated, and serum biochemistry values showed no abnormal changes from baseline by day 60. The Phase II trial [167] further demonstrated that HEV 239 is safe and immunogenic for humans, with 100% seroconversion after three doses [168]. The Phase III clinical trial [169] was conducted on 112,604 participants in China, wherein the vaccine was demonstrated to be safe and highly efficacious against HEV infection (100% seroconversion after three doses, 95% CI: 72.1–100). In fact, the HEV 239 vaccine induces a robust and persistent immune response with high efficacy against symptomatic and asymptomatic HEV infection.

Consequently, the HEV 239 vaccine was licensed and has been available in China since 2012 [170]. However, in its position paper about HE vaccination, the WHO concluded the need for more evidence for prequalification of the HEV 239 vaccine to gain global licensing, particularly concerning its efficacy and safety in outbreak conditions, pregnant women, patients with CLD, and immunosuppressed persons [7]. Recently, the first-ever mass vaccination effort for HEV outbreaks using this three-dose vaccine outside China was implemented in early 2022, targeting 27,000 residents of the Bentiu internment camp, South Sudan, who were aged 16–40 years [171]. This campaign was held despite challenges in organizing in the context of COVID-19, floods, rising food insecurity, and malnutrition. Interestingly, the high vaccination coverage is reassuring and suggests a community acceptance of the vaccine, even at a time of COVID-19-related vaccine hesitancy. This report may change the future position of the HEV 239 vaccine globally, favoring its worldwide licensing.

Regarding the third recent vaccine, the p179 vaccine proceeded to a phase I trial in 2017, compared with the HEV 239 vaccine. Results showed comparable safety and efficacy with a similar seroconversion as the control vaccine [163]. The authors of that study declared that a Phase II trial was in process, but no published data are available to date.

With an aim to enhance the immunogenicity and, in turn, the efficacy of the HEV vaccine, a recent study developed a fusion protein in a nonparticulate form that could improve the delivery and immunogenicity of E2 epitopes to elicit a better immune response. The study proposed fragment A of CRM197 as an intramolecular adjuvant carrier protein that can be fused to nonparticulate antigens that substantially enhanced the immunogenicity of the E2 protein [172].

### 6.3. Hepatitis E Vaccine in Elderly People

According to a recent study by Yu et al. in 2019, the majority (96.7%) of HEV 239-vaccinated elderly persons aged >65 years seroconverted at 1 month following the final dosage of this three-dose vaccine. At 7 months, 97.3% of them had satisfactory anti-HEV IgG levels. The vaccine was well tolerated with no documented adverse events in this cohort of elderly people [173].

### 6.4. Hepatitis E Vaccine in Pregnant Women

HEV infection in pregnant women may cause severe or fatal adverse events [174]. During the Phase III clinical trial of HEV 239 [169], 37 pregnant women in the HEV 239 vaccine group and 31 pregnant women in the control group were inadvertently vaccinated. The vaccine was well tolerated in these pregnant women without serious adverse events. Subsequently, the phase IV trial is currently ongoing, targeting the vaccination of approximately 20,000 women in the child-bearing period in rural Bangladesh (clinical trial: NCT02759991) [175]. Data that will be obtained from this ongoing study will significantly impact the ongoing process of global licensing of the HEV 239 vaccine.

### 6.5. Hepatitis E Vaccine in Immunocompromised Individuals

Solid organ transplant patients and persons with other immunosuppressive disorders, such HIV and rheumatological diseases, are more vulnerable to developing chronic HEV infection [155]. To date, there are no published trials on the use of an HE vaccine in this category of patients. Urgent large-scale studies are warranted to evaluate the safety and efficacy of HEV 239 in such immunocompromised patients.

### 6.6. Hepatitis E Vaccine in Patients with CLD

Patients with CLD have a higher risk of developing severe hepatitis E if infected [156]. This emphasizes the significance of adopting large-scale vaccination trials in such groups of patients, in concordance with the WHO recommendations [7]. A randomized controlled clinical study involving individuals who tested positive for HBsAg was conducted on HEV 239 vaccination in 2013. The study demonstrated high immunogenicity and good tolerability of the vaccine for both HBsAg-positive and HBV-negative individuals [176]. Furthermore, a phase IV study is being conducted on HEV 239 vaccination in people with chronic HBV in China (Clinical trials: NCT02964910), but the findings have not yet been disclosed.

### 6.7. Hepatitis E Vaccine in Children and Young Adolescents

In general, young individuals are at increased risk for infection in HEV-endemic developing countries [1,157]. In the recent outbreak in South Sudan in the early 2022, 422 (63%) of 670 suspected cases were younger than 16 years of age, including four of the reported nine deaths [171]. In fact, the HEV 239 vaccine was licensed only for individuals aged >16 years. This indicates the need to focus more on conducting extensive research, particularly in endemic areas, to reveal the wide-scale impact of vaccination on these people.

### 6.8. Hepatitis E Vaccine Durability

In the extended efficacy study of the HEV 239 vaccine in the Phase III clinical trial, long-term follow-up of up to 4.5 years demonstrated continuous efficacies of 93% (95% CI: 78.6–97.9%) among participants who received all three doses and 85% (95% CI: 67.1–93.3%) among those who received at least one dose. Furthermore, the trial demonstrated cross-protective efficiency across the common HEV1 and HEV4 genotypes in China [177]. However, data gaps remain regarding the longer protectivity, its efficacy when the scheduled doses are fewer than three, the impact of immunosuppressants on vaccine durability, and the need for booster doses.

### 6.9. Barriers of Global Hepatitis E Vaccination

To date, the HEV 239 vaccine is not prequalified by the WHO [7], which may preclude its use worldwide. Experience with HEV 239 vaccination, including the incidence of any negative events, should be recorded in all scenarios when it is used. Analysis of immunization in outbreak settings might yield useful information on the efficiency and safety of the vaccine as well as the rates of age-specific attacks. The other challenges to mass vaccination include transportation to endemic areas, regarding the need for cold chain storage and the fragility of the glass prefilled syringes. Nonetheless, the ongoing research on the real-time stability of the vaccine and the ability to transport it under the CTC setting may provide solutions for these challenges.

## 7. Frontiers of Hepatitis Vaccines

The positive impact of single-dose HA inactivated vaccine programs on FHF and liver transplantation in children has been increasingly demonstrated in Argentina, Brazil, and Russia [178,179,180]. In fact, the reported incidence of HAV decreased by 80–99% in all age groups. In Argentina, HAV had been the leading cause of FHF and liver transplantation in children in the prevaccination period. After the introduction of the universal single-dose childhood HA vaccination in 2005, there were no cases of liver failure or transplantation due to HA, demonstrating the positive impact of HA vaccination on these critical outcomes [178]. These data infer that the populations in middle-income countries may benefit the most from universal HA immunization programs.

Based on the latest WHO recommendations [181], HA vaccination should be added to national immunization schedules if there is an increase in HA incidence among older children, adolescents, or adults; if the endemicity changes from high to intermediate; or if the HA vaccination is cost effective. The WHO recommends a single-dose strategy for HA vaccines for children aged ≥12 years and for HAV outbreaks. Long-term data on the vaccine effectiveness, antibody persistence, and seroprotection indicate that the single-dose schedule is equivalent to the two-dose schedule in children, in addition to being less expensive and easier to implement [181].

Immunotherapy is a new player in the armamentarium against HBV. The major challenge in this strategy is the HBsAg-specific immune tolerance. There is an ongoing quest to breach this barrier by either breaking the immune tolerance and/or enhancing the immunogenicity of the vaccine. It was hypothesized that long-term suppression of HBsAg levels can induce HBsAg-specific B-cell responses [182,183]. Therefore, suppressing the high levels of circulating HBsAg should be a key factor for the immunotherapy of chronic HB infection [184]. Recently, a unique B-cell epitope-based particulate vaccine demonstrated effective suppression of HBsAg levels in mice. The candidate molecule was termed CR-T3-SEQ13 and was shown to promote a specific antibody response to the SEQ13 epitope in the HBV carrier mice and significantly suppress HBsAg and HBV DNA levels in the HBV-transgenic mice in a dose-dependent manner. In fact, based on a unique B-cell epitope, CR-T3-SEQ13 is a superior candidate for developing a therapeutic vaccine against HBV; it can be tested alone or in combination with HBV active drugs to decrease the levels of HBsAg. If successful, CR-T3-SEQ13-based immunotherapy will provide a novel strategy to achieve long-term suppression of HBsAg levels and improve the clinical outcome of chronic HB infection [185].

No approved HC vaccine to date, even in the most recent trials that utilized more than one vaccine, has reached its predetermined efficacy endpoint to prevent chronic HCV infection, irrespective of the induced T-cell response and relative safety of the used vaccines [186]. A breakthrough discovery was recently published by Torrents de la Peña et al. They presented for the first time the cryoelectron microscopic structure of the membrane-extracted full-length E1E2 heterodimer in complex with three broadly neutralizing antibodies. E1 and E2 viral glycoproteins are responsible for viral entry into the infected cells and are considered the major target for the immune system to produce neutralizing antibodies against HCV. The structure elucidates how the two subunits interact and describes three HCV key neutralizing epitopes. This understanding of E1 and E2 structure will significantly help in the discovery of more effective vaccine and may be a novel therapeutic option [187]. Donnison et al. recently attempted a bivalent viral vector vaccine in mice and found that it could induce both pangenotypic viral-specific B- and T-cell responses [188]. Moreover, Pihl et al. described a whole inactivated cell-culture-derived HC vaccine that was capable of inducing potent neutralizing antibodies [189]. *E. coli* heat-labile enterotoxin B subunit was also investigated recently by Feng et al., which demonstrated promising results in stimulating the secretion of IFN-γ and IL-4 as well as inducing humoral immune responses that could prevent viral entry [190]. The promising results of these recent trials need further exploration in future large-scale studies.

Nanoparticles have emerged as drug carriers that can improve drug bioavailability and efficacy via drug encapsulation. Recently, a veterinary vaccine based on nanoparticle technology “P206@PLGA” was introduced, targeting the zoonotic transmission of HEV genotype 4. This new vaccine is a swine-derived HEV4 HB-S4 strain. The immunogenic recombinant protein P206 was successfully synthesized using an *E. coli* expression system. A nanomaterial (poly lactic-co-glycolic acid, PLGA) was used to wrap the HEV ORF2 recombinant protein P206 to prepare nanoparticles (P206@PLGA) with a uniform size and stable quality. Experimental studies revealed its efficacy in animal immunization compared with conventional vaccines, with a potential safety profile both in vivo and in vitro [191]. This vaccine provides a new concept for HEV protection: to prevent the transmission of HEV in farms and protect susceptible populations.

## 8. Conclusions

The positive impact of the universal HA vaccination has been increasingly demonstrated in middle-income countries. Moreover, HA vaccination of travelers has substantially decreased the incidence of imported HAV-infected cases in high-income countries. The rate of HB vaccination increased globally to 85% in 2019. Universal infant HB vaccination had significantly decreased the incidence of HCC in high- and low-endemic areas. Recently, an HB vaccine using L-HBsAg that induces antibodies capable of neutralizing HBV with VEMs was stabilized. The recent discovery of E1 and E2 viral structures may significantly help in discovering more effective HCV vaccines. HEV 239 was the most extensively investigated HE vaccine and has been demonstrated to be safe and effective. The veterinary vaccine “P206@PLGA”, based on the nanoparticle technology, was recently introduced, targeting the zoonotic transmission of HEV genotype 4.

The WHO recommended a single-dose strategy for HA vaccines for children aged ≥12 years and for HAV outbreaks. The populations in middle-income countries may benefit the most from universal HA immunization programs (Table 2). The lower rate of African newborn babies receiving the HepB-BD vaccine and the reduction in HB infant vaccination during the COVID-19 pandemic may impede the WHO hepatitis elimination targets by 2030.

## Figures and Tables

**Table 1:** Current and past trials for hepatitis C vaccinations *.

NCT Number	Acronym	Status	Phases	Enrolment	Results
1. NCT02772003		Active, not recruiting	Phase I	33	No results available
2. NCT00857311		Completed	Phase I	17	Not effective
3. NCT00500747		Completed	Phase I	60	Safe and well tolerated, induced antibody, and lymphoproliferative responses
4. NCT01436357		Completed	Phase I Phase II	548	Produced T-cell response specific to HCV in 78% of participants, no serious adverse events, lowered peak HCV RNA level, but it did not prevent chronic HCV infection
5. NCT00445419		Completed	Phase I	30	No results available
6. NCT01701336	HCV004	Completed	Phase I	9	No results available
7. NCT01094873		Completed	Phase I	35	No results available
8. NCT01070407		Completed	Phase I	41	Vaccine induced antigen-specific CD8+ T cells in healthy human volunteers
9. NCT01296451		Completed	Phase I	55	Vaccine generated very high levels of both CD8(+) and CD4(+) HCV-specific T-cell responses targeting multiple HCV antigens
10. NCT00602784		Completed	Phase 2	66	No results available
11. NCT02568332		Completed	Phase I	20	No results available
12. NCT00393276		Completed	Phase I	29	No results available
13. NCT01055821	HCVac	Completed	Phase II	140	Vaccine was well tolerated and induced complete early virologic response when combined with interferon and ribavirin. Vaccine induced HCV-specific T-cell responses
14. NCT00124215		Completed	Phase II	48	No results available
15. NCT03119025		Completed	Phase I Phase II	10	Not effective
16. NCT00601770		Completed	Phase II	71	No results available
17. NCT02362217		Completed	Phase I	33	Vaccine was well tolerated and induced high-magnitude and broad T-cell responses
18. NCT00606086		Completed	Phase II	140	Not effective
19. NCT02027116	VGX-6150-01	Completed	Phase I	18	Enhanced HCV-specific T-cell responses, no significant side effects
20. NCT03674125		Completed	Phase I	32	No results available
21. NCT04318379		Recruiting	Phase I	30	No results available

* Some studies were excluded because they were withdrawn, had an unknown status, were terminated, or were not yet recruiting. HCV, hepatitis C virus.

**Table 2:** Limitations and recommendations for the protective vaccines against viral hepatitis.

Hepatitis Viruses	Available Vaccines	Vaccine Limitations	Future Perspective
Hepatitis A virus (HAV)	Inactivated vaccine	Generally requires a booster dose	Inclusion of universal hepatitis A vaccination in the national immunization schedules of middle-income countries A single-dose strategy for hepatitis A vaccines for children aged ≥12 years A single-dose strategy for hepatitis A vaccines for HAV outbreaks Continued monitoring of anti-HAV among individuals at high risk of infection Large-scale studies comparing short-term and long-term immune responses between inactivated and live attenuated vaccines
	Live attenuated vaccine	The majority of clinical trials are published in Chinese	
Hepatitis B virus (HBV)	Recombinant HB vaccine	Attenuated efficacy against HBV with vaccine-escape mutations (VEMs) 10% of vaccinated adults have low or no immune response Decreased vaccine compliance Booster dose vaccination may be needed	Global investment to scale-up infantile and hepatitis B birth dose vaccines in developing countries Wide-range adoption of the Heplisav-B^®^ vaccination in adults The combined use of S-HBs and L-HBs vaccines may neutralize strains with VEMs and strains of multiple HBV genotypes The use of two doses of 3A-HBV is suggested when accelerated immunization is indicated
	Heplisav-B^®^ vaccine	Limited efficacy in individuals with chronic obstructive pulmonary disease, renal failure, or cirrhosis	
	Tri-antigen HBV (3A-HBV) vaccine	Frequent serious adverse events compared with single-antigen HBV vaccine	
	Large hepatitis B surface antigen (L-HBsAg) vaccine	The neutralizing effects of induced antibodies depend on HBV genotypes	
Hepatitis C virus (HCV)	No available vaccine		
Hepatitis D virus (HDV)	No available vaccine. Yet, the immunization against HBV will also protect against HDV infection		
Hepatitis E virus (HEV)	HEV 239 vaccine	Not prequalified by the WHO Difficulties in transportation to endemic areas Requires three doses over 6-month duration to establish immunity	Large-scale clinical trials addressing long-term protectivity, efficacy when the scheduled doses are less than three, the impact of immunosuppressants, the need for booster doses, and the efficacy and safety in pregnant women, young adolescents, and patients with chronic liver disease (CLD). WHO legislation of HEV 239 vaccine for worldwide production particularly in endemic areas. Extended adoption of controlled temperature chain setting for delivering the vaccine to remote and difficult-to-access areas

## Data Availability

Not applicable.
